# MOS1 Negatively Regulates Sugar Responses and Anthocyanin Biosynthesis in Arabidopsis

**DOI:** 10.3390/ijms21197095

**Published:** 2020-09-26

**Authors:** Ning Zhang, Maike Wang, Jie Huang, Leiyun Yang, Zhixue Wang, Dianxing Wu, Xiaoli Shu

**Affiliations:** 1 State Key Laboratory of Rice Biology and Key Lab of the Ministry of Agriculture for Nuclear Agricultural Sciences, Institute of Nuclear Agricultural Sciences, Zhejiang University, Hangzhou 310029, China; 11216028@zju.edu.cn (N.Z.); 21716012@zju.edu.cn (M.W.); 21716002@zju.edu.cn (J.H.); 21316004@zju.edu.cn (Z.W.); 2 Plant Biology Section, School of Integrative Plant Science, Cornell University, Ithaca, NY 14853, USA; ly293@cornell.edu

**Keywords:** *Arabidopsis*, sugar signaling, anthocyanin biosynthesis, MOS1

## Abstract

Sugars, which are important signaling molecules, regulate diverse biological processes in plants. However, the convergent regulatory mechanisms governing these physiological activities have not been fully elucidated. MODIFIER OF snc1-1 (MOS1), a modulator of plant immunity, also regulates floral transition, cell cycle control, and other biological processes. However, there was no evidence of whether this protein was involved in sugar responses. In this study, we found that the loss-of-function mutant *mos1-6* (*mos1*) was hypersensitive to sugar and was characterized by defective germination and shortened roots when grown on high-sugar medium. The expression of *MOS1* was enhanced by sucrose. *Hexokinase 1*, an important gene involved in sugar signaling, was upregulated in the *mos1* mutant compared to wild-type Col-0 in response to sugar. Furthermore, the *mos1* mutant accumulated more anthocyanin than did wild-type Col-0 when grown on high-sugar concentration medium or under high light. MOS1 was found to regulate the expression of flavonoid and anthocyanin biosynthetic genes in response to exogenous sucrose and high-light stress but with different underlying mechanisms, showing multiple functions in addition to immunity regulation in plant development. Our results suggest that the immune regulator MOS1 serves as a coordinator in the regulatory network, governing immunity and other physiological processes.

## 1. Introduction

Sugars not only serve as energy sources in plants but also as hormone-like molecules in regulating many important physiological processes, including metabolism [1,2], seed germination [3], and biotic and abiotic stress responses [4,5]. Many sucrose-insensitive or -hypersensitive mutants have been screened to identify genes involved in sugar signaling [6,7,8,9]. By studying these mutants, it has been recognized that sugars have crosstalk with other signals, such as light [10], hormones [11,12], stresses [12], and nutrients [13,14]. Sugar signaling is usually triggered by glucose [15], although sucrose is the main type of sugar for systemic transport in plants [16].

Sugar signaling pathways are conserved in eukaryotes [17]. Hexokinases (HXKs), a group of identified glucose sensors, also govern glucose phosphorylation and regulate sugar responses [10]. In Arabidopsis, *HXK1* mutants are insensitive to glucose, and HXK1 has been reported to coordinate sugar, light, and hormones to control plant growth [10]. TREHALOSE-6-PHOSPHATE SYNTHASE (TPS), which is involved in the HXK-dependent glucose signaling pathway, catalyzes the biosynthesis of trehalose-6-phosphate from UDP-glucose and glucose-6-phosphate [18]. Overexpression of *AtTPS1* in Arabidopsis reduces the sensitivity to glucose [10,18]. G-protein-coupled receptors (GPCRs) identified in sugar signaling pathways can perceive sugar. Regulators of G-protein signaling (RGS) can activate the GTPase to drive G-protein into the inactive heterotrimer [19]. In Arabidopsis, glucose alters the interaction between G Protein Alpha Subunit1 (GPA1) and RGS1, consequently activating the hydrolysis of GTP and mediating sugar signal transduction [20]. Plant SNF1-RELATED KINASE (SnRK) proteins belong to a conserved SUCROSE-NONFERMENTING 1 (SNF1)/AMP-activated protein kinase (AMPK)/SnRK1 family, which plays important roles in metabolism regulation by sensing cellular energy charge [21,22,23]. SNF1 KINASE HOMOLOG 10 (AKIN10) and AKIN11, two Arabidopsis SnRK proteins, are reported to have important roles in sugar signaling pathways [12]. Recently, an evolutionarily conserved energy sensor TARGET OF RAPAMYCIN (TOR) complex has been demonstrated to link sugar signaling with meristem activation in Arabidopsis [24]. Sugar signals tightly coordinate the production and mobilization of sugars to regulate plant metabolism and development [25].

Moreover, environmental stresses would increase the accumulation of soluble sugars. Sucrose is necessary for producing anthocyanin, and sugars are closely associated with the regulation of anthocyanin biosynthesis [26,27]. As a class of secondary metabolites of flavonoids, anthocyanins are widely found in plants [28]. Anthocyanins absorb light in a certain wavelength range and play roles in the prevention of photoinhibition [29]. Anthocyanins are also antioxidants and confer multiple tolerances against abiotic and biotic stresses, including cold, UV, pathogens, and insects, by ROS scavenging [30]. The biosynthesis of anthocyanin in plants begins with the conversion of phenylalanine into coumarate-CoA by phenylalanine ammonia lyase (PAL), cinnamate-4-hydroxylase, and 4-coumarate: CoA ligase, which are common steps shared by many secondary metabolic pathways [31]. The subsequent biosynthesis processes can be divided into early and late stages. In the early biosynthesis stage, coumarate-CoA is catalyzed consecutively by chalcone synthase (CHS), chalcone isomerase (CHI), flavanone-3-hydroxylase (F3H), flavonoid-3′-hydroxylase (F3′H), and flavonoid-3′5′-hydroxylase (F3′5′H) to form three types of dihydroflavonols [32]. In the late biosynthesis stage, dihydroflavonol 4-reductase (DFR), anthocyanidin synthase (ANS), and UDP-glucose flavanol 3-O-glucosyl transferase are specific enzymes mediating anthocyanin biosynthesis from dihydroflavonols [32]. The *FLAVONOL SYNTHASE* (*FLS*) gene encodes a flavonol synthase that catalyzes the formation of flavonols from dihydroflavonols [33]. Genes participating in these two steps are called anthocyanin biosynthesis genes (*ABGs*). MYB-type transcription factors (TFs), basic helix–loop–helix (bHLH)-type TFs, and WD40-repeat TFs form MBW complexes to regulate the biosynthesis of anthocyanins [34]. The expression of genes encoding the MBW complex subunits is strongly induced by such factors as sugars, hormones, and environmental stresses, which subsequently activate the transcription of *ABG*s [35,36].

Currently, emerging evidence has shown that sugars are involved in immunity [4,37]. MODIFIER OF snc1-1 (MOS1), a modulator of plant immunity, positively regulates the NLR genes *SUPPRESSOR OF npr1-1* and *CONSTITUTIVE 1* (*SNC1*). MOS1 is responsible for the autoimmunity phenotype in *bonzai1* (*bon1*) and *snc1* [38,39] by binding to the promoter region of *SNC1* to activate its expression by interacting with TCP TFs [40]. MOS1 was also found to regulate floral transition by interacting with Suppressor of FRIGIDA 4, a transcriptional activator of *Flowering Locus C* [39]. Moreover, MOS1 plays roles in endoreduplication regulation [39]. These results show that MOS1 acts as an intermediary regulator to coordinate growth and defense in a complicated network. CONSTITUTIVE EXPRESSION OF PR GENES5 (CPR5) is a regulator of growth and defense and acts in a resistance pathway dependent on Non-expresser of Pathogenesis-Related genes 1 (NPR1), a sugar hypersensitive mutant *hypersenescence1* allelic to the *cpr5* mutant [41]. However, there is no evidence of whether MOS1 is involved in sugar responses. 

Mutant *mos1* has been reported to have delayed flowering, increased ploidy level, and changed rosette size and other mutant phenotypes [39,40]. A previous study revealed that MOS1 antagonized MAD1 activity by interacting with MAD2 in endoreduplication regulation [40]. *MAD2* loss-of-function mutants have defects in early seedling development, and these defects can be rescued by exogenous sugars [42]. Therefore, it is possible that exogenous sugar treatment also affects *mos1* seedling development. To characterize the roles of MOS1 in plant development, in this study, we investigated the responses of *mos1* mutants, wild-type Col-1, and *mos1* complementation lines *#1* and *#2–9* under different sugar concentrations, including the germination rate, the expression of *MOS1*, a sugar-responsive gene *Subunit 3 of ADP-Glucose Pyrophosphorylase* (*APL3*) [43], and other genes involved in the sugar response pathway, i.e., *HXK1* and *TPS1* in the HXK1-dependent pathway [10,18], *RGS1* and *GPA1* in the RGS pathway [20,44], *AKIN10* and *AKIN11* in the SNF1-RELATED KINASE1 pathway [22,23,45,46], and *SUGAR-INSENSITIVE 3* encoding an E3 ligase in an independent sugar-response pathway [47]. We found that the *MOS1* knockout mutant *mos1* exhibited hypersensitive responses to sugar. We hypothesized that MOS1 might participate in sugar signaling pathways and other processes related to sugar signaling. The *HXK1* gene involved in sugar signaling pathways showed enhanced expression in the *mos1* mutant in response to sugar. As sucrose is known as a positive factor in the accumulation of anthocyanin pigments [2] and high light stress can also boost the biosynthesis of anthocyanin [27], we speculated that MOS1 might also be involved in sugar- and light-induced anthocyanin biosynthesis. Then, we analyzed anthocyanin accumulation in wild-type Col-0 and *mos1*, *#1*, and *#2–9* grown on medium supplemented with different concentrations of sugar and grown in the same soil medium but under different light intensities and found that MOS1 is involved in the regulation of anthocyanin biosynthesis triggered by sugar and light by affecting the expression of *ABGs* and *FLS*. This finding suggests that MOS1 has multiple roles in organizing sugar signaling and immune responses, thereby functioning as a coordinator in developmental, biotic, and abiotic stress responses.

## 2. Results

### 2.1. mos1 Mutant Was Hypersensitive to Sugar during Early Seedling Development

**Figure 1:**
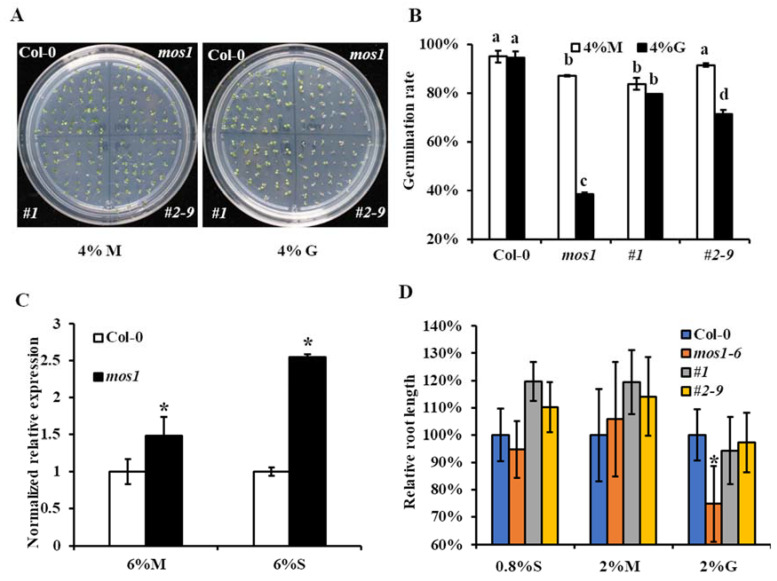
Hypersensitive phenotypes of *mos1* in response to sugars. (**A**) Representative images of the germination of Col-0 and *mos1*, *#1*, and *#2-9* grown on half-strength MS medium with 4% mannitol (M) or 4% glucose (G). (**B**) The germination rates of Col-0 and *mos1*, *#1*, and *#2–9* grown on half-strength MS medium with 4% M or 4% G. Different letters above the bars indicate significant differences (one-way ANOVA/Bonferroni *p* < 0.001). (**C**) qRT-PCR analysis of *APL3* expression in Col-0 and *mos1* seedlings grown on half-strength MS medium with 6% M or 6% S. Quantification was normalized to *ACTIN2*. Error bars indicate standard error (SE) of two independent biological replicates. The asterisk indicates a significant difference compared with the corresponding Col-0 (one-way ANOVA/Bonferroni *p* < 0.001). (**D**) Relative root lengths of 7-d-old Col-0 and *mos1*, *#1*, and *#2–9* seedlings grown on half-strength MS medium with 0.8% sucrose (S), 2% M or 2% G.

When *mos1* mutant and wild-type Col-0 seeds were sown on medium supplemented with exogenous sugars, there was no difference in the germination rates of the *mos1* mutant from that of wild-type Col-0 when sown on half-strength Murashige and Skoog (MS) medium supplemented with 0.8% sucrose (*w/v*) (normal medium; Figure S1). However, when seeds were sown on half-strength MS medium supplemented with 4% glucose, the germination rate of the *mos1* mutant was reduced to 40% (Figure 1A,B), while the germination rate of wild-type Col-0 was 94% (Figure 1A,B). A similar germination phenotype was observed when seeds were sown on medium supplemented with 6% sucrose (Figure S1). However, when *mos1* and wild-type Col-0 seeds were sown on half-strength MS medium supplemented with 4% mannitol (equimolar concentrations of glucose), no significant difference in germination rates was observed between them (Figure 1A,B), indicating that the defective germination of *mos1* sown on medium supplemented with 4% glucose or 6% sucrose was not caused by osmotic stress but by sugars.

Furthermore, we investigated the expressions of *APL3* under different sugar treatments. The expression level of *APL3* in *mos1* was similar to that of wild-type Col-0 when seedlings were grown on half-strength MS supplemented with 6% mannitol (Figure 1C), while it was 160% higher in *mos1* when grown on half-strength MS supplemented with 6% sucrose (Figure 1C). These results were consistent with a previous study [48] and indicated that the *mos1* mutant was sensitive to sugar.

To confirm whether the absence of *MOS1* is responsible for sugar hypersensitivity in the *mos1* mutant, we generated the MOS1 rescue construct p*MOS1::MOS1:GFP* and obtained two independent complementation lines, p*MOS1::MOS1:GFP mos1–6 #1* (*#1*) and p*MOS1::MOS1:GFP mos1–6 #2–9* (*#2–9*) (Figure S2). These two lines partially rescued the defect germination rate of *mos1* grown on medium supplemented with sugar (Figure 1A,B and Figure S1), confirming that the knockdown of *MOS1* is responsible for the defective germination rate and contributes to the sugar hypersensitivity of *mos1*.

As high-sugar treatment also affected root elongation [25], we analyzed the root lengths of the wild-type Col-0 and *mos1*, *#1*, and *#2–9* seedlings grown on medium containing different concentrations of sugar for 7 d. The root lengths of *mos1* were comparable to the wild-type when seedlings were grown on normal medium or half-strength MS with 2% mannitol (Figure 1D). However, when seedlings were grown on half-strength MS with 2% glucose, the roots of *mos1* were shorter than those of wild-type Col-0 (Figure 1D). Complementation lines *#1* and *#2–9* both showed normal root elongation, as with the wild-type (Figure 1D). This finding confirmed that the shortened root in *mos1* was due to the loss of function of *MOS1*.

### 2.2. Expression of MOS1 Is Induced by Sucrose

**Figure 2:**
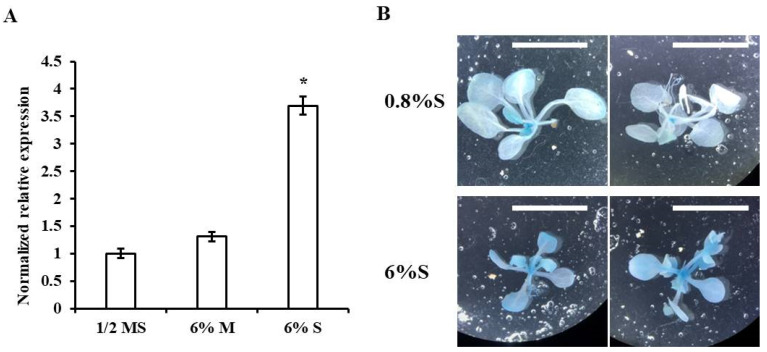
*MOS1* expression in response to sugar. (**A**) qRT-PCR analysis of *MOS1* expression in 10-d-old Col-0 seedlings grown on half-strength MS medium with 0.8% sucrose (S), 6% mannitol (M), or 6% S. Quantification was normalized to *ACTIN2*. Error bars indicate the standard error (SE) of two independent biological replicates. The asterisk indicates a significant difference compared with seedlings grown on half-strength MS medium with 0.8% sucrose (one-way ANOVA/Bonferroni *p* < 0.001). (**B**) Representative images showing the *β*-glucuronidase activity of the p*MOS1::GUS* line grown on half-strength MS medium with 0.8% S or 6% S. Scale bars = 1 cm.

As *mos1* showed hypersensitivity to sugars, to explore the role of MOS1 in sugar signaling pathways, the expression of the *MOS1* gene in wild-type Col-0 under different concentrations of sucrose was investigated. Compared to the seedlings grown on normal medium, the expression level of *MOS1* in seedlings grown on medium supplemented with 6% sucrose increased by 270% (Figure 2A). To elucidate the expression pattern of *MOS1* in response to exogenous sucrose, the transgenic plants *pMOS1:GUS* harboring the *β*-glucuronidase (GUS) reporter gene under the promoter and the first exon of *MOS1* [39] were used. Histochemical analysis showed that the expression of *GUS* driven by the *MOS1* promoter was related to the developmental stages of leaves and sucrose concentrations. When seedlings were grown on normal medium, strong GUS signals were detected in the emerging tissues, but only notably weak GUS signals were detected in the mature tissues. However, when seedlings were transferred to medium containing 6% sucrose, the intensity of the GUS signal was stronger, with obvious GUS signals being detected in the mature tissues (Figure 2B). These results showed that the expression of *MOS1* is promoted by exogenous sucrose.

### 2.3. MOS1 Affects the Expression of HKX1 in Response to Sugar

**Figure 3:**
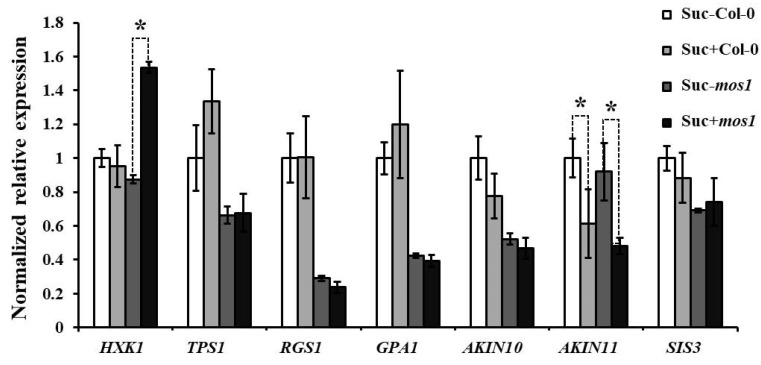
Sugar related gene expression analysis in Col-0 and *mos1*. qRT-PCR analysis of the expression of genes involved in sugar-response pathways in Col-0 and mos1 grown on half MS at 3 hours after 6% sucrose (Suc+) or 6% mannitol (Suc-) treatment. Quantification was normalized to ACTIN2. Error bars indicate SE of two independent biological replicates. The asterisks indicate a significant difference between Suc+ and Suc- in each genotype (Student’s *t*-test *p* < 0.001).

According to the qRT-PCR results, the expression of seven genes in several well-established sugar-response pathways was unchanged in both wild-type Col-0 and the *mos1* mutant in response to sucrose, except for *HKX1* and *AKIN11* (Figure 3). The transcription of *AKIN11* was downregulated by sucrose in both wild-type Col-0 and *mos1* mutants, which made it difficult to determine whether the transcription change was associated with MOS1. However, the transcription of *HKX1* was significantly upregulated by sucrose in the *mos1* mutant and unchanged in wild-type Col-1 (Figure 3). This finding suggested that the *mos1* mutation may influence the HKX1-dependent sugar response pathway.

### 2.4. MOS1 Represses Anthocyanin Biosynthesis Induced by Sugar and High-Light Stress

**Figure 4:**
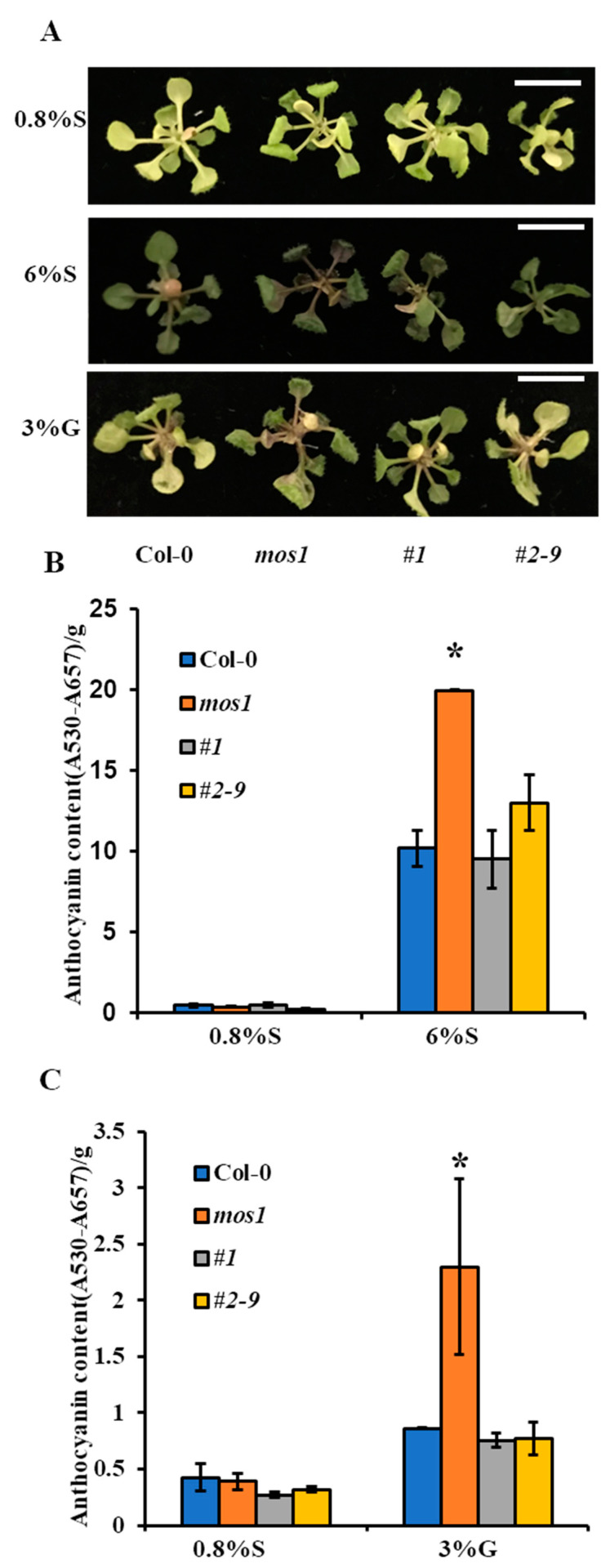
Anthocyanin accumulation induced by sugar in different lines. (**A**) Representative images of phenotypes of Col-0 and *mos1*, *#1*, and *#2–9* grown on half-strength MS medium with 0.8% sucrose (S), 6% S, or 3% G. Scale bars = 1 cm. (**B**) Anthocyanin content in the seedlings of Col-0 and *mos1*, *#1*, and *#2–9* grown on half-strength MS medium with 0.8% S or 6% S. (**C**) Anthocyanin content in the seedlings of Col-0 and *mos1*, *#1*, and *#2–9* grown on half-strength MS medium with 0.8% S or 3% G. Error bars indicate SE of two independent biological replicates. The asterisks indicate significant differences compared with Col-0 under the same treatment (one-way ANOVA/Bonferroni *p* < 0.001).

When seedlings were grown on normal medium, mutant *mos1* had comparable anthocyanin content to wild-type Col-0. However, when seedlings were grown on medium supplemented with 3% glucose or 6% sucrose, *mos1* accumulated 2.5- and 2-fold more anthocyanin than wild-type Col-0, respectively (Figure 4). In addition, complementation lines *#1* and *#2–9* accumulated similar amounts of anthocyanin pigments to wild-type Col-0 under all conditions (Figure 4).

**Figure 5:**
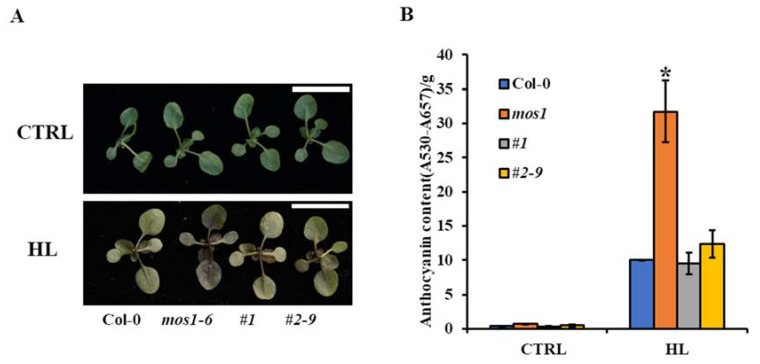
Anthocyanin accumulation under high light in different lines. (**A**) Representative images of the phenotypes of Col-0 and *mos1*, *#1*, and *#2–9* grown in soil after high-light treatment. Scale bars = 1 cm. (**B**) Anthocyanin content in the seedlings of Col-0 and *mos1*, *#1*, and *#2–9* grown in soil under high light (HL) or normal light (as CTRL). Error bars indicate the standard deviation (SD) of three measurements. The asterisk indicates a significant difference compared with Col-0 under the same treatment (one-way ANOVA/Bonferroni *p* < 0.001).

Additionally, the accumulation of anthocyanin in response to high light in wild-type Col-0 and *mos1*, *#1*, and *#2–9* were analyzed concurrently. As shown in Figure 5, the anthocyanin content in *mos1* was similar to that in wild-type Col-0 under normal conditions. After high-light treatment, *mos1* accumulated anthocyanin pigments three times those in wild-type Col-0, while the contents of anthocyanin pigments in complementation lines *#1* and *#2–9* were similar to those in wild-type Col-0 (Figure 5). This finding indicated that MOS1 could repress anthocyanin biosynthesis induced by sucrose and high-light stress, although the mechanisms governing the effect warrant further analysis.

### 2.5. MOS1 Affects the Expression of Genes Related to Anthocyanin Biosynthesis in Response to Sugar and High Light

**Figure 6:**
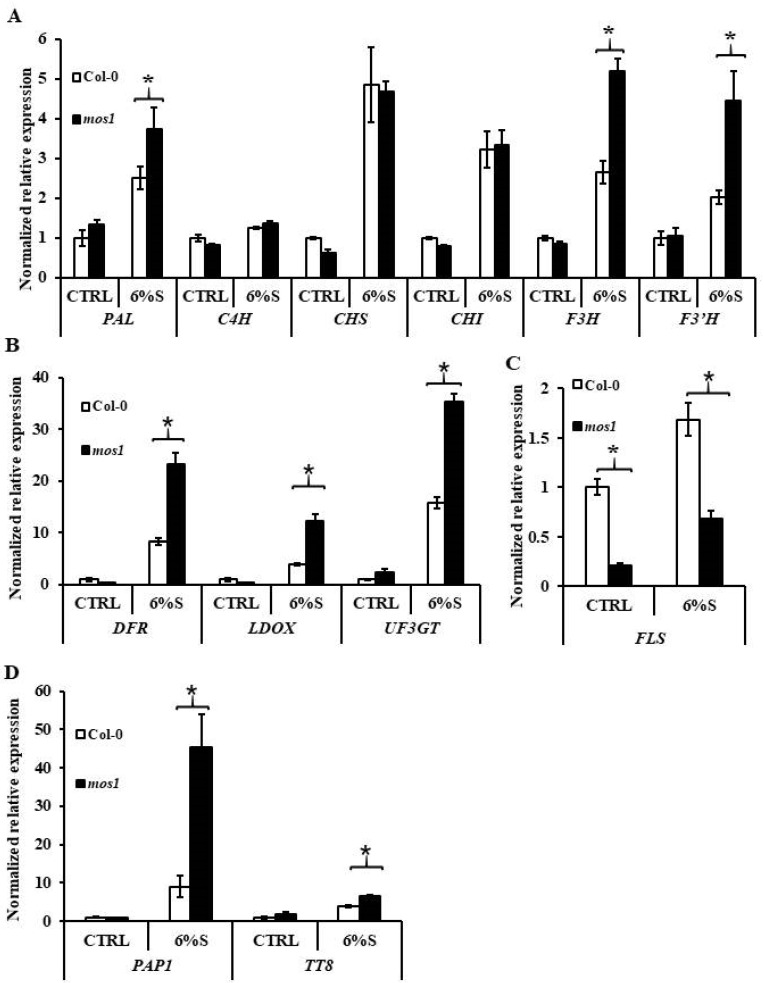
Expression analysis of anthocyanin biosynthesis genes in response to sucrose. qRT-PCR analysis of the expression of early *ABG*s (**A**), late *ABG*s (**B**), *FLS* (**C**), and TFs (**D**) in 10-d-old Col-0 and *mos1* after 6% mannitol (CTRL) or 6% sucrose (6%S) treatment. Quantification was normalized to *ACTIN2*. Error bars indicate SE of two independent biological replicates. The asterisks indicate significant differences compared with the corresponding Col-0 (one-way ANOVA/Bonferroni *p* < 0.001).

To discover the molecular regulatory mechanisms of MOS1 on anthocyanin accumulation, the transcription of six early *ABGs* (*PAL*, *C4H*, *CHS*, *CHI*, *F3H*, *F3′H*), three late *ABGs* (*DFR*, *ANS*, *LDOX*), *FLS*, and two components of MBW complex, *PAP and TT8*, were analyzed. After treatment with 6% sucrose, the expression levels of *PAL* and *F3H* and *F3′H* in *mos1* were 50%, 96%, and 109% higher than those in wild-type Col-0, respectively, and the expression levels of *DFR*, *LDOX*, and *UF3GT* in *mos1* were 180%, 207%, and 124% higher than those in wild-type Col-0, respectively (Figure 6A,B). However, the expression levels of *DFR*, *LDOX*, and *UF3GT* in *mos1* treated with 6% mannitol were similar to those in wild-type Col-0 (Figure 6A,B). Moreover, after sucrose treatment, the expression level of *FLS* was 60% lower in *mos1* than in wild-type Col-0, although its expression was also lower upon treatment with 6% mannitol (Figure 6C). Correspondingly, after treatment with 6% sucrose, the transcript levels of *PAP1* and *TT8* were 400% and 62% higher than those in wild-type Col-0, respectively, but there was no difference after treatment with 6% mannitol (Figure 6D).

**Figure 7:**
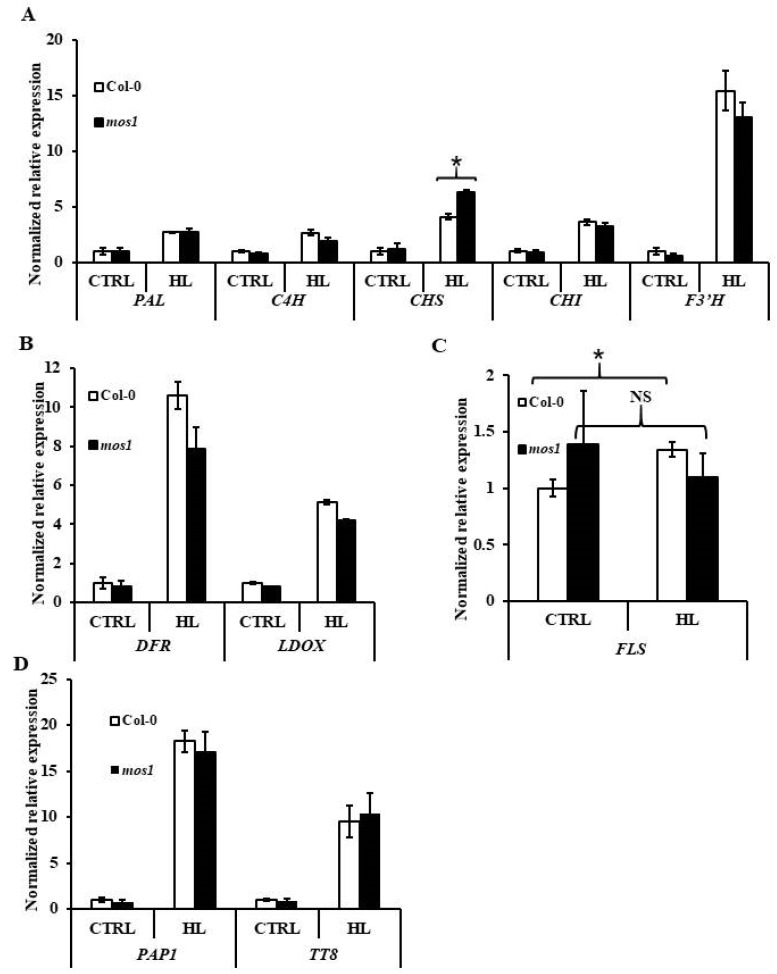
Expression analysis of anthocyanin biosynthesis genes in response to light. qRT-PCR analysis of the expression of early *ABG*s (**A**), late *ABG*s (**B**), *FLS* (**C**), and TFs (**D**) in 14-d-old Col-0 and *mos1* seedlings grown on soil under normal (CTRL) or 24 h high-light (HL) treatment. Quantification was normalized to *ACTIN2*. Error bars indicate SE of two independent biological replicates. The asterisks indicate significant differences compared with the corresponding Col-0 (one-way ANOVA/Bonferroni *p* < 0.001). NS, not significant.

The expression of most *ABG*s in *mos1* was equivalent to that in wild-type Col-0 under both normal and high-light conditions, except for *CHS*. The expression abundance of *CHS* after high-light treatment in *mos1* was 54% higher than that in wild-type Col-0 (Figure 7A,B). In addition, the expression of *FLS* in *mos1* after high-light treatment was similar to that under normal light, while it was increased in wild-type Col-0 after high-light treatment (Figure 7C). This finding indicates that MOS1 affects the expression of *FLS*. Moreover, the expression of *PAP1* and *TT8* was similar in the wild-type Col-0 and *mos1* under both normal and high-light conditions (Figure 7D). Although the responses of these genes to high light and sucrose were different in *mos1*, we can still conclude that MOS1 might regulate the accumulation of anthocyanin under sugar and light treatment by influencing the expression of some *ABGs* but through different regulatory mechanisms.

## 3. Discussion

Sugar signaling plays important roles in plant development and abiotic and biotic stress responses [7]. In this study, we found that the absence of the *MOS1* gene function caused intense responses to sugars, as characterized by a reduced germination rate and shortened roots. Correspondingly, the expression of the glucose-responsive marker gene *APL3* was increased (Figure 1), and *MOS1* could respond to exogenous sucrose (Figure 2). This finding indicated that MOS1 was a negative regulator of sugar responses and that there might be transcriptional feedback to control the responses within a certain range. The higher expression of *HXK1* in response to sucrose in *mos1* than in wild-type Col-0 (Figure 3) suggested that MOS1 may influence sugar responses by regulating the transcriptional level of *HXK1*. HXKs have been identified as glucose sensors in many plant species, and recently, HXK1 was discovered to have multiple functions [9], i.e., promoting anthocyanin biosynthesis in apple by stabilizing a bHLH TF [49]. In agreement with this finding, *mos1* accumulated more anthocyanin than wild-type Col-0 when exposed to exogenous sugars (Figure 4).

In addition to sugars, anthocyanin biosynthesis is triggered by multiple stresses [2,5,27,50]. The overaccumulation of anthocyanin pigments in the *mos1* mutant under sugar and high-light stress compared to wild-type Col-0 (Figure 4 and Figure 5) indicated that MOS1 negatively regulates anthocyanin biosynthesis. Sugar activated the expression of several *ABG*s (Figure 6) [43], which was more pronounced in *mos1* (Figure 6). As *mos1* exhibited increased sensitivity to sugars, the overaccumulation of anthocyanin pigments could be a consequence of the enhanced sugar response. Sugars also enhanced the expression of *FLS* [2,33] (Figure 6C); as a hypersensitive mutant, *mos1* should have a higher expression of *FLS*. However, *mos1* had a significantly lower expression level of *FLS* than did wild-type Col-0 (Figure 6C), and the lower expression level of *FLS* led to dihydroflavonol accumulation as substrates for subsequent anthocyanin biosynthesis. This finding indicates that MOS1 has other mechanisms independent of sugar signals in regulating anthocyanin biosynthesis.

Moreover, the regulatory mechanisms of MOS1 in anthocyanin biosynthesis under sugar and light stresses might be different. Upon high-light treatment, only the expression of *CHS* in the *mos1* mutant was higher than that in the wild-type, while under 6% sucrose, several *ABGs* but no *CHS* had different expression levels between the *mos1* mutant and wild-type Col-0 (Figure 6 and Figure 7). Moreover, the expression of *FLS* in CTRL under sugar treatment was different from that under light treatment (Figure 6 and Figure 7), which might be due to the pretreatment in the dark before transport to medium containing different sugars compared to no pretreatment before transfer to chambers with different light intensities. Additionally, the expression of *FLS* was not induced by high-light treatment in *mos1* (Figure 7C). All these pieces of evidence indicate the specific function of MOS1 in the transcriptional regulation of *FLS*. A MOS1-interacting protein [40], TCP15, represses anthocyanin biosynthesis under high light [51], suggesting that TCP15 and MOS1 might also be involved in anthocyanin biosynthesis as well as immune responses. TCP15 affects the expression level of *PAP1*, *TT8*, and *DFR* under high light [51], while MOS1 showed no influences on *PAP1*, *TT8*, and *DFR* under high light (Figure 7). That might be because the regulation mechanisms of TCP15 and MOS1 on anthocyanin synthesis do not overlap completely, just like in immune responses [40]. Additionally, we used different sampling time points from Vialo et al. [51], while the effects of TCP15 had been found to be related to the irradiation time [51]. However, MOS1 showed negative regulations on the expression of *PAP1*, *TT8*, and *DFR* under 6% sucrose (Figure 6). The expressions in the early part of the high-light treatment and the global gene expression changes with RNA-seq will be conducted in further studies, which will be beneficial for obtaining a better understanding of the regulation of MOS1 and the interactions of MOS1 and TCP15 on anthocyanin biosynthesis combined with genetic analysis. 

Similar to anthocyanin biosynthesis, plant defense responses are affected by many factors, such as hormones, sugars, and light [52,53,54,55]. Recently, there has been increasing evidence supporting the contribution of sugar signals to plant immune responses. HXK1 plays positive roles in immune regulation, and the glucose phosphorylation capacity of HXK1 has been found to be essential for cell death and defense responses in the *MIPS* (*myo*-inositol 1-phosphate synthase) mutant [37]. MOS1 also plays positive roles in immunity, but the *mos1* mutant has normal defense responses [37,39,40]. Thus, there is a possibility that the enhanced *HXK1* expression in *mos1* may be a compensation mechanism to maintain proper immune responses, and MOS1 might be the convergent regulator involved in the sugar-immunity regulation network.

Some studies also suggested that anthocyanin could take part in immunity in plants, but the precise underlying mechanism remains uncharacterized [56,57]. 

As MOS1 showed functions in anthocyanin accumulation (Figure 4 and Figure 5), it may be worthwhile to identify convergent regulators in anthocyanin biosynthesis and immune response crosstalk, which may provide new insights into the coordinated network between immunity and other physiological processes.

## 4. Materials and Methods

### 4.1. Plant Materials and Growth Conditions

*Arabidopsis thaliana* Col-0 ecotype (available in Arabidopsis information service, N1092), the *mos1-6* mutant (*mos1*) derived from Col-0 by gamma irradiation, and pMOS1:GUS created in a previous study [38] were graciously provided by Dr. J Hua (Cornell University, USA). Two complementary lines, p*MOS1::MOS1: GFP mos1–6#1* (*#1*) and p*MOS1::MOS1: GFP mos1–6 #2–9* (*#2–9*), were created and identified in this study by Z.W. and L.Y. All plants were grown in chambers with 50% humidity at 22 °C and under 12-h light (light intensity: 150 μmol m^−2^ s^−1^) and 12-h dark.

To ensure that the plants grew normally, half-strength MS with 0.8% (*w/v*) sucrose was used as normal medium. For sugar treatment, 10-d-old seedlings grown on normal medium were transferred to the dark for 24 h to reduce intercellular sugar. After that step, the medium was replaced by half-strength MS medium with 3% (*w/v*) glucose, 6% (*w/v*) sucrose, 3% (*w/v*), and 6% (*w/v*) mannitol for an additional 3 h under light.

For the high-light treatment, plants were grown in soil under 150 μmol m^−2^ s^−1^ light (normal) for 14 d. Then, some plants were transferred to chambers with a light intensity of 450 mol m^−2^ s^−1^ (high light). Seedlings treated for 1 d were used for RNA isolation, and seedlings treated for 3 d were used to analyze the anthocyanin content.

### 4.2. Plasmid Construction and Generation of Transgenic Plants

A genomic fragment of the entire *MOS1* coding region (without stop codon) and the 2680-bp sequence upstream of the ATG start codon were amplified by PCR from genomic DNA isolated from Col-0. The PCR product was cloned into the pDONR222 vector by BP reactions (Invitrogen, 11789020) and then cloned into the binary vector pGWB550 [58] to create *pMOS1:MOS1:GFPCOM*. The constructed vector was introduced into *mos1* using *Agrobacterium tumefaciens* GV3101. Transgenic plants were selected on plates with hygromycin.

### 4.3. Germination Assay and Root Length Measurement

All seeds, harvested and stored identically, were sown on normal medium and medium containing 4% glucose or 4% mannitol. All plates were incubated at 4 °C for 2 d and then placed in a growth chamber for 7 d. The germination rate was scored by cotyledon greening. At least 50 seeds for each genotype were used for each independent biological repeat, and two repeats were conducted.

For root length measurement, seedlings were grown vertically on normal medium and medium supplemented with 2% glucose or 2% mannitol for 7 d. Images were captured by a digital camera, and the root lengths were calculated by ImageJ.

### 4.4. GUS Staining

To analyze GUS activity in response to sugar, *pMOS1::GUS* transgenic lines [30] were grown on the indicated medium. The seedlings were dipped into chilled 90% acetone and then stained in 100 mM sodium phosphate buffer (pH 7.2) containing 1 mM 5-bromo-4-chloro-3-indolyl-β-glucuronic acid, 2 mM K_3_Fe(CN)_6_, 2 mM K_4_Fe(CN)_6_, 10 mM EDTA, and 0.1% (*v/v*) Triton X-100 at 37 °C. After staining, 70% (*v/v*) ethanol was used to remove the chlorophyll. Images were recorded by a LEICA S9 stereoscope.

### 4.5. Measurement of Anthocyanin Content

Fresh seedlings grown on the indicated medium or after light treatment were used for measuring anthocyanin content. Leaf tissues of 20 mg were homogenized in 0.6 mL of methanol–HCl (1%, *v/v*) and then incubated at 4 °C for 1 d. After centrifugation, 0.4 mL chloroform and 0.4 mL ddH_2_O were added to the supernatant and vortexed vigorously. Then, the samples were centrifuged, and the absorbance of the supernatant was measured at 530 and 657 nm. Relative anthocyanin concentrations were calculated with the equation anthocyanin content = (A530-A657)/fresh weight (g).

### 4.6. RNA Extraction and Quantitative Real-Time PCR Analysis

Total RNA was isolated from plants with RNAiso Plus (Takara, Shiga, Japan, 9108), according to the manufacturer’s instructions. cDNA was synthesized from 2 µg RNA by a PrimeSpcript^TM^ RT reagent Kit with a gDNA Eraser Kit (Takara, Shiga, Japan, RR047). Quantitative RT-PCR was performed with a Bio-Rad CFX96™ Real-Time System (Bio-Rad, Hercules, USA) using TB Green Premix Ex Taq^TM^ II (Tli RNaseH Plus; Takara, Shiga, Japan, RR820). Primers for RT-PCR are listed in Table S1. Two independent biological replicates were performed.

## 5. Conclusions

We provide evidence that the immune regulator MOS1 represses sugar responses and anthocyanin biosynthesis in Arabidopsis, possibly at the transcriptional level. Our findings highlight the involvement of MOS1 in sugar signaling. In the future, identifying MOS1 genetic interacting regulators and studying the regulation of MOS1 in sugar and hormone signaling may not only help to characterize the roles of MOS1 in specific biological processes but also elucidate the mechanism governing the balance of growth and defense.

## Supplementary Materials

Supplementary materials can be found at https://www.mdpi.com/1422-0067/21/19/7095/s1. Table S1: Primers sequences for RT-PCR in this study. Figure S1: Representative images of the germination of Col-0 and *mos1*, *#1*, and *#2–9* grown on 1/2 MS medium with 0.8% sucrose, 6% Man, or 6% Suc. Figure S2: RT-PCR analysis of MOS1 expression in Col-0 and *mos1*, *#1*, and *#2-9*.

## Abbreviations

ABGs	anthocyanin biosynthesis genes
AKIN	SNF1 kinase homolog
APL3	Subunit3 of ADP-Glucose Pyrophosphorylase
ANS	anthocyanidin
bHLH	basic helix–loop–helix
bon1	bonzai1
CHI	chalcone isomerase
CHS	chalcone synthase
DFR	dihydroflavonol
F3H	flavanne-3-hydroxylase
FLS	flavonol synthase
GUS	β-glucuronidase
HEX1	Hexokinases 1
HL	high-light
MAD1	mitotic arrest deficient1
MOS1	modifier of snc1-1
MS	Murashige and Skoog
PAL	Phenylalanine ammonia lyase
PAP1	Purple acid phosphatase 1
SNC1	suppressor of npr1-1, constitutive 1
TFs	transcription factors
TT8	Transparent testa8
