# Supplementary material for: MOS1 Negatively Regulates Sugar Responses and Anthocyanin Biosynthesis in Arabidopsis
Source: Int J Mol Sci. 2020 Sep 26;21(19):7095. doi: 10.3390/ijms21197095 (PMC7584024; doi:10.3390/ijms21197095)
Supplement: Supplementary file 1 [file ijms-21-07095-s001.pdf]

Supplementary Information

MOS1 Negatively Regulates Sugar Responses and Anthocyanin Biosynthesis in Arabidopsis

Table 1. Primers sequences for RT-PCR in this study.

ACTIN2-qRT-F	GACCTTTAACTCTCCCGCTA
ACTIN2-qRT-R	GGAAGAGAGAAACCCTCGTA
APL3-F	CACACGGATGTTTCTTGGGA
APL3-R	GGTAACTATCCGCTCCTAAC
MOS1-qRT-F	GCATGGGATTCGAACTCGC
MOS1-qRT-R	GTCAAGCATTGGAGGTCTGAAC
HXK1-F	GAATCCAGGCGAACAGATTCTTGAG
HXK1-R	GTGCATAGCCGACATGTGAGGAG
TPS1-F	GGTGTCACAAAGGGAGCTGC
TPS1-R	CATCTTCGTCCTTCCCCAAG
RGS1-F	TTTCCTCCCCCTTGTTTTGTT
RGS1-R	ATGAAGGCCTGCAACTGGG
GPA1-F	TGAACGTTTGCGAGTGGTTC
GPA1-R	GGCGCCGTGTTCTGGTAATA
AKIN10-F	TCCCCGTGAAATAATGACGG
AKIN10-R	CATACCATCTGCGCTGCTGT
AKIN11-F	TCCTATGCGCACACCTGAAG
AKIN11-R	TCCAAGAGCCCATTTTCGAT
SIS3-F	TTGTTGACAATGGTCTTGCTTC
SIS3-R	AAGCCCACAGAAATGGATACAG
PAL-F	CCAAAAACGGTGTCGCACT
PAL-R	GCTTCCGAATATTCCGGCGTTAA
C4H-F	GGAGAAATCAACGAGGACAATGTTC
C4H-R	CCACTCGATAGACCACAATGTTGT
CHS-F	CCGACCTCAAGGAGAAGTTCAAG
CHS-R	GCATGTGACGTTTCCGAATTGT
CHI-F	GAATCTATCCCGTTCTTCCGTGAA
CHI-R	GGCAGTTTCATTGTCACCTTGATAA
F3H-F	AGGAGCGTTTGTCGTCAATCTC
F3H-R	GCATTCTTGAACCTCCCATTGC
F3'H-F	CAGACATCGCTCAGCTTCCTTA
F3'H-R	GGTGGATGAAGCCTGAAATTCTCTT
DFR-F	CCTTATCACCGCGCTCTCT
DFR-R	TGTCCTTGTCTTATGATCGAGTAATGC
LDOX-F	TCAATTTGGCCTAAGACACCAAGT
LDOX-R	TCGCTAGCAAACGAAGACACTT
UF3GT-F	CAACTGGTTTTCCGTTTCTGGTT
UF3GT-R	GCTTCCTCGACGGTTGATACAC
FLS-F	CTTCCGGGATCATCGTCATCATC
FLS-R	GCCCTATGCTCCACACTCTTATACT
TT8-F	ATGGAGGAAGGTGGAAACTATTC
TT8-R	GGCAAACGATGATTGGATGTAAG
PAP1-F	TTCCTGAAGCGACGACAACA
PAP1-R	AGCAAACCTATACACAAACGCA

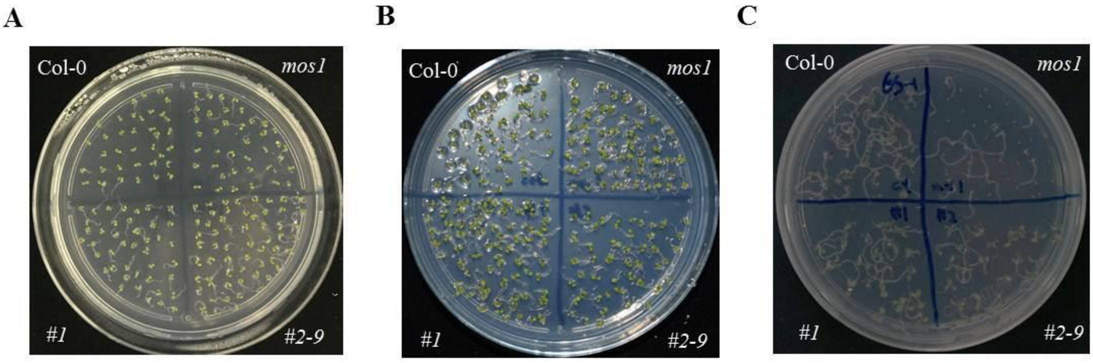

Figure S1. Representative images of the germination of Col-0, *mos1*, *#1* and *#2-9* grown on 1/2 MS medium with 0.8% sucrose (**A**), 6% Man (**B**)or 6% Suc (**C**).

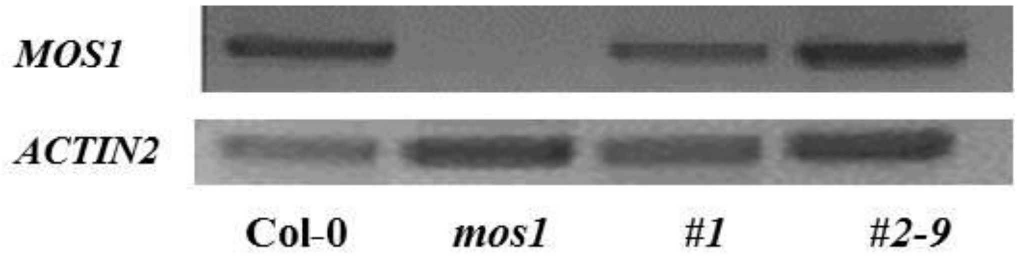

Figure S2. RT-PCR analysis of MOS1 expression in Col-0, mos1, #1 and #2–9. Only the target bands were cropped from the original gel.
